# Factors Affecting User Acceptance in Overuse of Smartphones in Mobile Health Services: An Empirical Study Testing a Modified Integrated Model in South Korea

**DOI:** 10.3389/fpsyt.2018.00658

**Published:** 2018-12-12

**Authors:** Seo-Joon Lee, Mun Joo Choi, Mi Jung Rho, Dai-Jin Kim, In Young Choi

**Affiliations:** ^1^Research Institute of Health Science, Korea University, Seoul, South Korea; ^2^Department of Medical Informatics, The Catholic University of Seoul, Seoul, South Korea; ^3^Department of Biomedicine & Health Sciences, College of Medicine, The Catholic University of Korea, Seoul, South Korea; ^4^Catholic Institute for Healthcare Management and Graduate School of Healthcare Management and Policy, The Catholic University of Korea, Seoul, South Korea; ^5^Department of Psychiatry, Addiction Research Institute, Seoul St. Mary's Hospital, College of Medicine, The Catholic University of Korea, Seoul, South Korea; ^6^Department of Psychiatry, Seoul St. Mary's Hospital, College of Medicine, The Catholic University of Korea, Seoul, South Korea

**Keywords:** smartphone overuse, m-Health, TAM, UTAUT, acceptance

## Abstract

Smartphones have become crucial in people's everyday lives, including in the medical field. However, as people become close to their smartphones, this leads easily to overuse. Overuse leads to fatigue due to lack of sleep, depressive symptoms, and social relationship failure, and in the case of adolescents, it hinders academic achievement. Self-control solutions are needed, and effective tools can be developed through behavioral analysis. Therefore, the aim of this study was to investigate the determinants of users' intentions to use m-Health for smartphone overuse interventions. A research model was based on TAM and UTAUT, which were modified to be applied to the case of smartphone overuse. The studied population consisted of 400 randomly selected smartphone users aged from 19 to 60 years in South Korea. Structural equation modeling was conducted between variables to test the hypotheses using a 95% confidence interval. Perceived ease of use had a very strong direct positive association with perceived usefulness, and perceived usefulness had a very strong direct positive association with behavioral intention to use. Resistance to change had a direct positive association with behavioral intention to use and, lastly, social norm had a very strong direct positive association with behavioral intention to use. The findings that perceived ease of use influenced perceived usefulness, that perceived usefulness influenced behavioral intention to use, and social norm influenced behavioral intention to use were in accordance with prior related research. Other results that were not consistent with previous research imply that these are unique behavioral findings regarding smartphone overuse. This research identifies the critical factors that need to be considered when implementing systems or solutions in the future for tackling the issue of smartphone overuse.

## Introduction

### Background

Smartphones have become crucial in our everyday lives, and they affect all sectors from business, to communication, and even to medicine. However, as people become close to their smartphones, they are easily led to engaging in overuse without even acknowledging it. Unfortunately, excessive use sometimes leads to disorders, such as fatigue due to lack of sleep (1), depressive symptoms and social relationship failure (2), and job interruption (3), and in the case of adolescents, it can hinder academic achievement (4). Regarding overuse, the International Classification of Diseases now categorizes “gaming disorder” as a mental illness. This is the first time that the consequences of using digital and mobile devices have been so designated, which makes it more meaningful (5).

In our previous research, we proposed the Smartphone Overdependence Management System (SOMS), which is a management system, and solution for overdependence on smartphones (6). The SOMS was designed to deliver overuse prevention, diagnosis, and treatment services that are based on scientific evidence, which was mostly collected by the developed SOMS smartphone background software application (“app”). The idea was to use the concept of mobile technology that has been extensively applied and widely successful in other healthcare systems (7–11) to aid self-control and support behavioral change in such a way that overuse is controlled.

The neurophysiological impacts of the devices themselves are also important to consider. There have been several studies that show the biological effects of various waveforms emanating from mobile devices, which cause activation of the sympathetic nervous system (SNS) through oxidative stress and other defined mechanisms (12, 13). When the SNS is activated, behavioral change is more difficult to achieve, especially when biochemical conversion pathways are initiated (e.g., adrenalin). This means that the device environment is also a barrier to forms of self-control. Thus, there is a difference between information system use regarding a hardwired desktop and accessible laptop and mobile devices. That is, mobile versions have a unique additional problem in that physical bioeffects are caused by the device itself.

The SOMS is currently in the stage of being implemented in a practical way in order to facilitate the remote monitoring of smartphone overuse by users. Most importantly, for successful intervention, factors affecting the user acceptance of such a system or solution must be evaluated beforehand. Specific services can then be developed or modified as effective solutions that best suit the needs of the user. The Technology Acceptance Model (TAM) and Unified Theory of Acceptance and Use of Technology (UTAUT) model have been frequently used to analyze the factors for acceptance, which will be specifically explained in the study design section in the methods.

### Research Related to the Acceptance of Smartphone Overuse Monitoring Apps

The definition of smartphone overuse in this article includes all addictive activities, such as excessive use of the internet, playing games, logging on to messenger services, or accessing virtual communities to the extent that the person neglects positive areas of life, which is similar to the definition of Billieux et al. (14). Although there have been many programs and research studies of interventions for smartphone overuse, there have been few that have used smartphone apps for smartphone overuse intervention. Lin et al. incorporated app-recorded data into the psychiatric criteria for the diagnosis of smartphone addiction and examined the predictive ability of the app-recorded data for the diagnosis of smartphone addiction (15). In addition, Lee et al. proposed a comprehensive information and communications technology (ICT) system called the Smartphone Addiction Management System for smartphone addiction management and verification. The idea was to monitor the users' app usage together with GPS location and internet access location. Other than these few studies, smartphone mobile apps have mostly targeted other risk factors, such as cannabis addiction management (16), smoking cessation (17), and so on.

### Goals and Objectives

Therefore, the aim of this study was to investigate the determinants of the users' intention to use m-Health for smartphone overuse interventions. The research results are expected to identify not only the critical factors to consider when implementing the SOMS, but also to provide successful mainstream guidelines for future systems or solutions that tackle the issue of smartphone overuse, for example, apps that are being released that have screen-time monitors.

## Methods

### Study Design

The research model of this paper was fundamentally based on the TAM and UTAUT models. The TAM is a widely accepted and influential model that predicts users' perceptions or acceptance of information system use (18–20). The UTAUT is the latest derivative of the TAM. The TAM has been constantly developed by researchers, which has led to the creation of many variants, such as the Extended TAM, Psychosocial TAM, and Integrated TAM (21).

**Figure 1:**
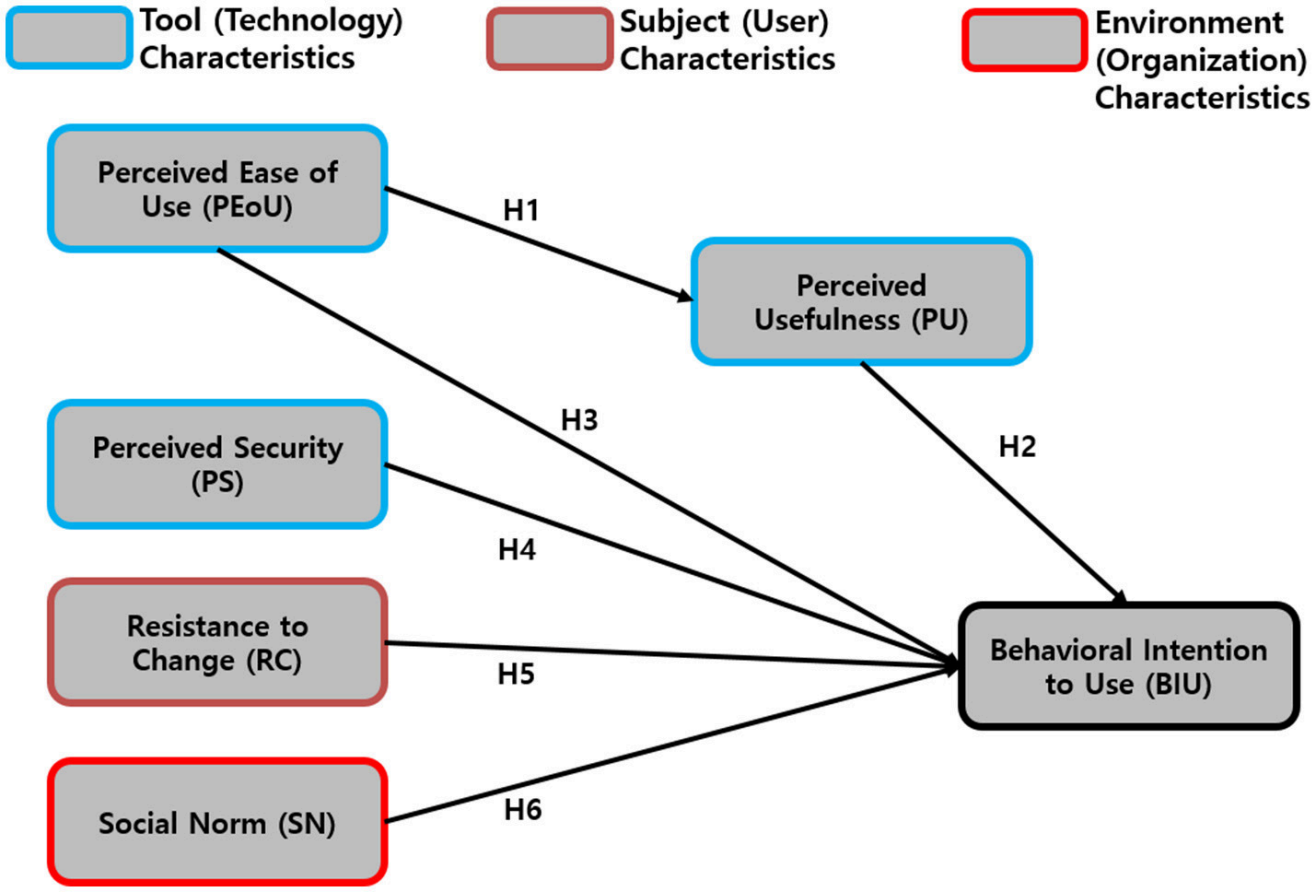
The Modified Technology Acceptance Model and Unified Theory of Acceptance and Use of Technology Model for Smartphone Overuse Intervention Apps.

Although this research was based on the TAM and UTAUT, we modified these models by converging, excluding, or including some important variables that were identified as appropriate in the case of smartphone overuse intervention by smartphone apps, as shown in Figure 1.

The TAM basically proposes two main factors that determine the users' behavioral intention to use (BIU), which are its perceived usefulness (PU), and perceived ease of use (PEoU). It was also acknowledged by the researchers that, at times, PU acts as a mediator between PEoU and BIU; thus, this was taken into account.

In this model, perceived security (PS) was added, since personal security issues in network services have been a threat to many services including in the field of healthcare, which obtains sensitive private information. Altogether, PU, PEoU, and PS comprise the tool characteristics (which represent the technology).

Other factors, such as resistance to change (RC) and social norm (SN), were added, with each representing the subjects' characteristics (representative of the users) and environmental characteristics. The research hypotheses were tested in relation to the model proposed above and they are shown in Table 1.

**Table 1:** Summary of the proposed research hypotheses.

**Hypothesis**		**Models used to test the hypotheses**
H1	PEoU has a positive direct effect on PU	Original TAM, Extended TAM, Psychosocial TAM, Integrated TAM, UTAUT
H2	PU has a positive direct effect on BIU
H3	PEoU has a positive direct effect on BIU
H4	PS has a positive direct effect on BIU
H5	RC has a positive direct effect on BIU
H6	SN has a positive direct effect on BIU

*H, hypothesis; TAM, technology acceptance model; UTAUT, unified theory of acceptance and use of technology*.

### Studied Population and Sample

The studied population consisted of 400 randomly selected smartphone users aged from 19 to 60 years who were from a social survey institution panel (which was evenly pooled domestically), which was to be extrapolated to the smartphone users of South Korea. Those who had provided written informed consent prior to the survey were accepted. Participants were briefly informed about the SOMS before the survey (shown in the first page of our survey). Participants who were not adults were excluded because of the possibility that they would not understand the research. A brief but sufficient explanation was given about the research before the survey. Participants older than 60 years of age were excluded because they are not accustomed to smartphones.

The size of the sample population was selected based on the following criteria. According to the July 2017 statistics, ~9 out of 10 adults use smartphones (population *N* = 37,454,121). For reliability within the 95% confidence interval, the appropriate recommended sample size was 385. Considering the possibility of missing values, we recruited 15 more people, adding up to a total of 400.

Only participants who used smartphones for at least 1 h a day were selected. Among them, half (*n* = 200) were selected based on the condition that they had the experience of using similar smartphone usage monitoring/measuring app(s). The other half (*n* = 200) were selected based on the condition that they did not have any experience of using any similar smartphone usage monitoring/measuring app(s). These groups were evenly selected to prevent a bias of friendliness toward smartphone apps.

This research study's procedures were performed in accordance with the Declaration of Helsinki. The Institutional Review Board of the Catholic University of South Korea, St. Mary's Hospital (MC17QESI0076), approved the study.

### Variables

**Figure 2:**
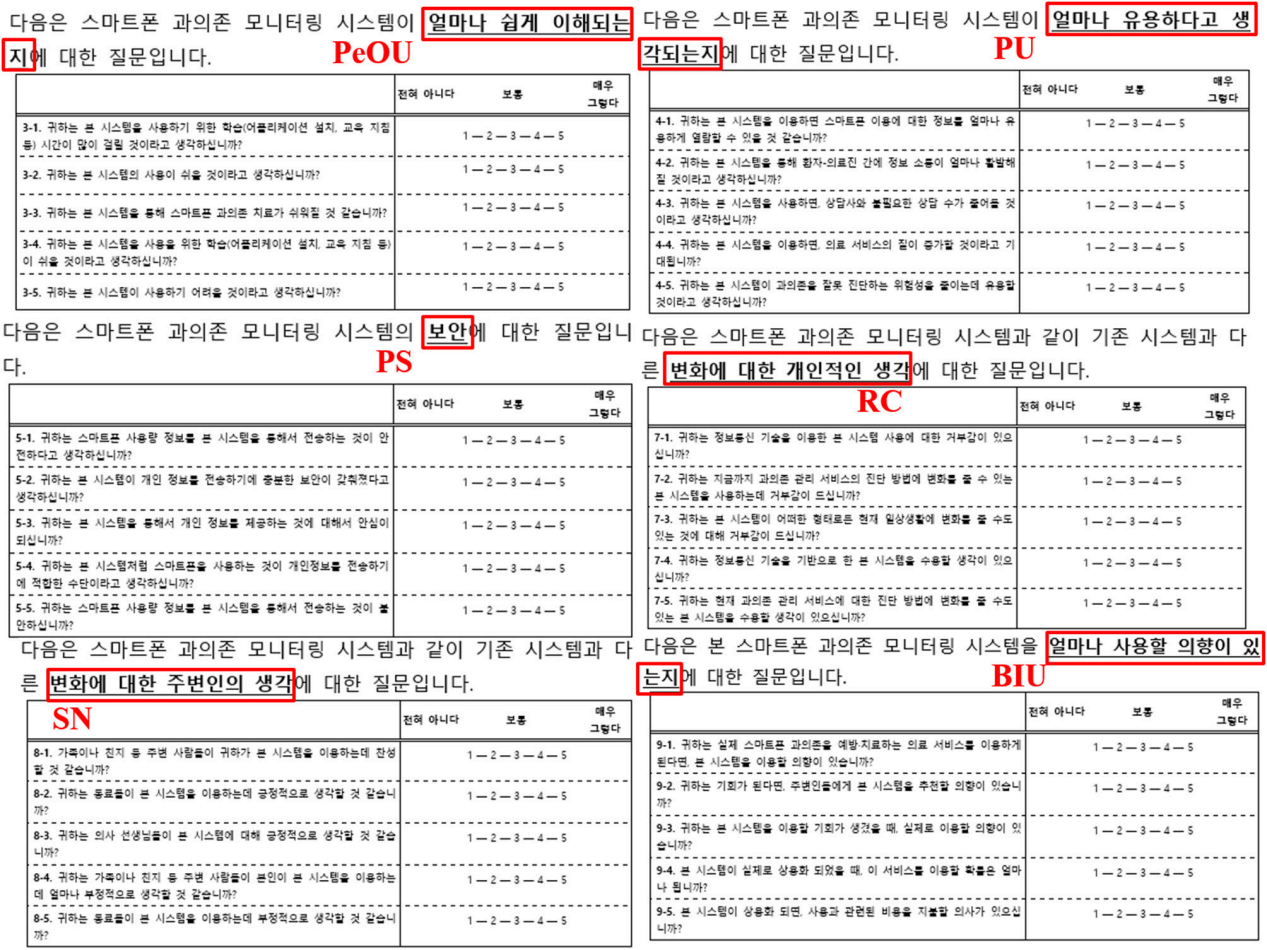
Screenshots of the Survey Taken Regarding PeOU, PU, PS, RC, SN, and BIU.

In this research, the acceptance (or BIU) of a smartphone overuse monitoring app among smartphone users was the dependent latent variable. The term “smartphone overuse monitoring system” was defined at the beginning of the questionnaire. All questions, except for those that required short-answer questions or socio-demographic information, captured responses through a five-point scale. This five-point Likert scale ranged from “strong negative,” “negative,” “neutral,” “strong,” to “strong positive.” Five survey items were measured for each of the PEoU, PU, PS, RC, SN, and BIU latent variables based on prior TAM- and UTAUT-related research (21–24), but they were modified to suit the current research study's theme. Screenshots of our survey are presented in Figure 2 (the Korean to English translation is shown in red).

The PEoU items included “Do you think it will take long to learn the smartphone overuse monitoring system?” (PEoU1), “Do you think that it will be easy to use such a system?” (PEoU2), “Do you think that smartphone overuse treatment will become easier due to this system?” (PEoU3), “Do you think that learning how to use this system will be easy?” (PEoU4), and “Do you think that this system will be hard to use?” (reverse question, PEoU5).

The PU items included “How useful do you think this system will be when learning to control the use of smartphones?” (PU1), “Do you think that doctors and patients will become closer during overuse treatment due to this system?” (PU2), “Do you think that by using this system, needless overuse consulting sessions will decrease?” (PU3), “Do you think that by using this system, the quality of service regarding smartphone overuse treatment will improve?” (PU4), and “Do you think that this system will contribute to reducing misdiagnosis of actual smartphone usage time?” (PU5).

The PS items included “Do you think it is safe to transfer your personal smartphone usage information via this system?” (PS1), “Do you think that this system has sufficient security to transmit personal information?” (PS2), “Would you feel safe when transmitting personal information via this system?” (PS3), “Do you think that using smartphones is a reliable way to transmit personal information?” (PS4), and “Would you feel uncomfortable when transmitting smartphone usage information via this system?” (reverse question, PS5) (Supplementary Table 1).

The RC items included “Do you find this ICT applied system repulsive?” (RC1), “Do you find this system, which may change the current method of diagnosing smartphone overuse, repulsive?” (RC2), “Do you find this system, which could bring some change to your way of life, repulsive?” (RC3), “Are you willing to accept this ICT applied system?” (reverse question, RC4), and “Are you willing to accept this system, which is expected to change the current method of diagnosing smartphone overuse?” (RC5).

The SN items included “Do you think your family or friends will agree with you using this system?” (SN1), “Do you think your friends or coworkers will think positively about you using this system?” (SN2), “Do you think that doctors will be positive about this system?” (SN3), “How negatively do you think your family or friends will think about you using this system?” (SN4), and “How negatively do you think your friends or coworkers will think about you using this system?” (SN5).

Lastly, the BIU items included “If you were to use a smartphone overuse prevention or treatment service, would you consider using this system?” (BIU1), “If the opportunity arises, would you recommend this system?” (BIU2), “If the opportunity arises for you to use this system, would you use it?” (BIU3), “If this system was implemented as an actual service, what are the chances that you will use it?” (BIU4), and “If this system was implemented as an actual service, would you be willing to pay the right amount of fees to use this system's service?” (BIU5).

### Statistical Analyses

Descriptive statistical analysis was conducted to summarize the overall socio-demographic results. Then, correlation analysis was performed to measure the magnitude of the associations between the independent latent variables and the dependent latent variable for each hypothesis (i.e., H1, H2, H3, H4, H5, and H6). Structural equation modeling (SEM) was also conducted to test each hypothesis. All statistical analyses were performed using SAS 9.3 with a 95% confidence interval. All values were rounded up to the second decimal place.

## Results

### Socio-Demographic Results

The socio-demographic results are shown in Table 2. Fifty percent of the participants were men (*n* = 200) and 50% were women (*n* = 200). Forty percent were 19 to 29 years old (*n* = 160), 40% were 30 to 39 years old (*n* = 160), and 20% were over 40 (*n* = 80). The most used app was SNS, with a percentage of 48.75 (*n* = 195).

**Table 2:** Socio-demographic results of the participants.

**Characteristics**		***N***	**Percentage**
Gender	Male	200	50.0
	Female	200	50.0
Age group (years)	19–29	160	40.0
	30–39	160	40.0
	Over 40	80	20.0
Most Used App During the Past Year^a^	SNS	195	48.75
	Web Surfing	104	26.0
	Game	33	8.25
	Entertainment	28	7.0
	Shopping	10	2.5
	Taking Photos	6	1.5
	Others	23	6
Location^b^	Seoul	128	32.0
	Gyeonggi/Incheon	127	31.75
	Chungcheong	27	6.75
	Jeonla	26	6.5
	Gyeongsang	82	20.5
	Others	10	2.5
Education	Middle School or Lower	43	10.75
	High School	65	16.25
	Graduate School	253	63.25
	Ph.D. or Above	39	9.75
Occupation^c^	White-Collar	161	40.25
	Administrative Position	25	6.25
	Service	34	8.5
	Technical Professional	48	12.0
	Student	60	15.0
	Others	72	18.0
Monthly Salary (U.S. Dollars)^d^	< 1.8 Thousand	65	16.25
	1.8 Thousand ≤ to < 2.7 Thousand	82	20.5
	2.7 Thousand ≤ to < 3.7 Thousand	73	18.25
	3.7 Thousand ≤	180	45.0
Perceived Socio-Economic Status	Low	160	40.0
	Middle	230	57.5
	High	10	2.5
Total		400	100

a *Other apps included those for health, diet, transportation, finance, weather, and so on*.

b *Other locations included Gangwon and Jeju*.

c *Other occupations included agricultural and blue-collar*.

d *Approximate value converted from Korean won to U.S. dollars*.

A plurality of respondents lived in Seoul (32%, *n* = 128). Most respondents were at least graduate school students (63.25%, *n* = 253) and the most common occupation was white-collar (40.25%, *n* = 161). The most common monthly salary group was 3.7 thousand dollars or more per month (45%, *n* = 180). Regarding the perceived socio-economic-status, middle class was the most common (57.5%, *n* = 230).

### Variables Measured and Psychometric Properties

Based on the Kaiser-Meyer-Olkin test, confirmatory factor analysis was not acceptable for some theoretical constructs; therefore, they were excluded. Only acceptable theoretical constructs were chosen, which are shown in Table 3 (an average of 0.88). The Bartlett's test results also showed a chi-square value of 4133.91, with 231 degrees of freedom, and a *p*-value lower than 0.0001. In addition, the Cronbach's alpha values were acceptable for all constructs among the acceptable constructs of the confirmatory factor analysis (0.75 to 0.88), except for PEoU (0.65); however, this was adequate based on past findings (25). Table 3 shows the mean, standard deviation (*SD*), Cronbach's alpha, factor loading, construct reliability, and average variance extracted of all the constructs.

**Table 3:** Theoretical Constructs and Psychometric Properties of the Measures.

**Construct**	**Mean ±*SD***	**Cronbach's alpha**	**Factor loading**	**Construct reliability**	**Average variance extracted**
Perceived ease of use		0.65		0.722	0.474
PEoU2	3.35 ± 0.84		0.75		
PEoU4	3.2 ± 0.81		0.692		
PEoU5	2.49 ± 0.88		0.433		
Perceived usefulness		0.82		0.861	0.556
PU1	3.29 ± 0.83		0.591		
PU2	3.31 ± 0.86		0.708		
PU3	3.21 ± 0.87		0.624		
PU4	3.21 ± 0.83		0.771		
PU5	3.25 ± 0.88		0.749		
Perceived security		0.87		0.888	0.666
PS1	2.68 ± 0.92		0.734		
PS2	2.48 ± 0.92		0.867		
PS3	2.28 ± 0.98		0.843		
PS4	2.54 ± 0.95		0.743		
Resistance to change		0.75		0.776	0.635
RC1	2.5 ± 0.95		0.734		
RC3	2.56 ± 0.89		0.816		
Social norm		0.77		0.844	0.647
SN1	3.09 ± 0.77		0.729		
SN2	3.12 ± 0.76		0.855		
SN3	3.21 ± 0.83		0.596		
Behavioral intention to use		0.88		0.895	0.632
BIU1	3.13 ± 0.94		0.818		
BIU2	3.13 ± 0.87		0.779		
BIU3	3.18 ± 0.9		0.844		
BIU4	2.94 ± 0.94		0.803		
BIU5	2.41 ± 1.03		0.635		

*SD, standard deviation; PEoU, perceived ease of use; PU, perceived usefulness; PS, perceived security; RC, resistance to change; SN, social norm; BIU, behavioral intention to use*.

### Correlation Analysis Between the Theoretical Constructs

The correlation analysis between all the theoretical constructs is shown in Table 4. All results were statistically significant within a 95% confidence interval. The constructs' correlations ranged from 0.69 to 0.82.

**Table 4:** Correlation analysis between the theoretical constructs.

	**PEOU**	**PU**	**BIU**	**PS**	**RC**	**SN**
PEOU	**0.689**					
PU	0.553	**0.746**				
BIU	0.317	0.637	**0.795**			
PS	0.174	0.408	0.436	**0.816**		
RC	0.243	0.258	0.391	0.214	**0.797**	
SN	0.425	0.533	0.704	0.451	0.416	**0.805**

*PEoU, perceived ease of use; PU, perceived usefulness; BIU, behavioral intention to use; PS, perceived security; RC, resistance to change; SN, social norm. Bold values represents Pearson's Correlation Coefficients*.

### Fit Indices of the Measurement Models and Their Acceptable Ranges

The measurement models' fit indices, including the acceptable thresholds, are shown in Table 5. The chi-square/degrees of freedom (χ^2^/*df*) was 2.895 (≤ 3.00 recommended), the goodness-of-fit index (GFI) was 0.884 (≥ 0.90 recommended), the adjusted GFI index (AGFI) was 0.851 (≥ 0.90 recommended), the non-normed fit index (NNFI) was 0.865 (≥ 0.90 recommended), the comparative fit index was 0.906 (≥ 0.90 recommended), and the root mean square residual was 0.063 (≤ 0.08 recommended). Although the values of the GFI, AGFI, and NNFI were slightly lower than recommended, it was concluded that all fit indices were acceptable and supported a reasonable fit assumption.

**Table 5:** Fit indices of the measurement models and their acceptable ranges.

**Model-fit index**	**Recommended value**	**Scores**
Chi-square/degrees of freedom	≤ 3.00	2.895
Goodness-of-fit index	≥0.90	0.884
Adjusted goodness-of-fit index	≥0.90	0.851
Non-Normed fit index	≥0.90	0.865
Comparative fit index	≥0.90	0.906
Root mean square residual	≤ 0.08	0.063

### Measurement Model and Structural Model

**Figure 3:**
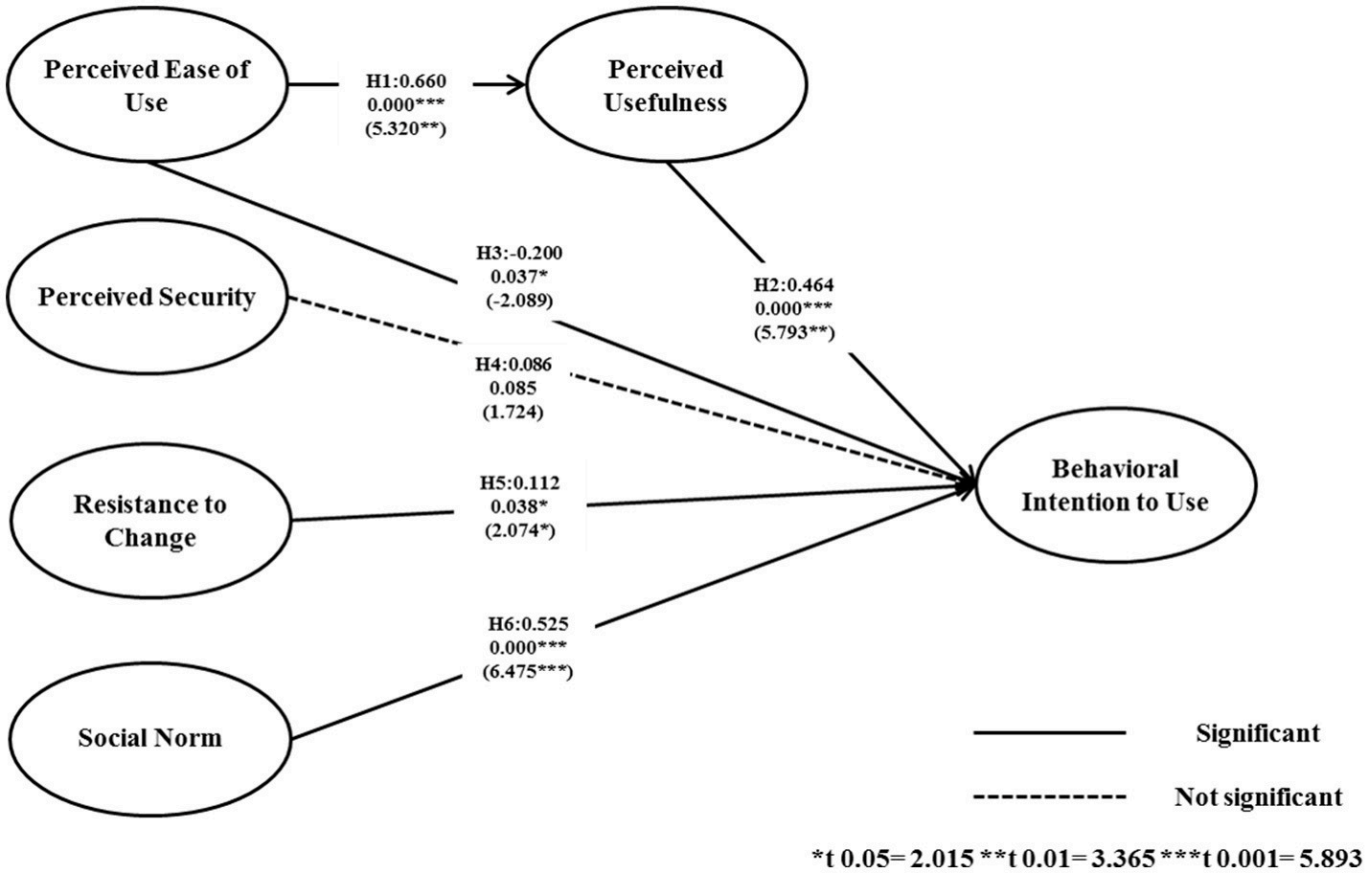
Structural Model and Standardized Regression Coefficients. H: hypothesis.

The results of the SEM are presented in this section. All constructs were in accordance with the results shown in Table 3. All socio-demographic information was adjusted. Figure 3 shows the results of the structural model. The findings supported H1, H2, H3, H5, and H6, but not H4. Therefore, PEoU had a very strong direct positive association with PU (H1, positive correlation), and PU had a very strong direct positive association with BIU (H2, positive correlation). However, in the case of H3, PEoU had a direct negative association with BIU, rather than a positive association (H3, negative correlation). RC had a direct positive association with BIU (H5, positive correlation) and, lastly, SN had a very strong direct positive association with BIU (H6, positive correlation).

## Discussion

### Comparison With Prior Work

Although examined in other fields (and considering the fact that factor analysis of the perception of smartphone overuse monitoring apps is scarce), the result that Perceived Ease of Use is a significant determinant of Perceived Usefulness is consistent with the findings of numerous previous research studies (19, 26–28). In accordance with the discussion in these studies, it can be concluded that, in the case of smartphone overuse monitoring apps, the friendliness of the user interface can critically affect the treatment compliance among users.

In addition, the current study's results that show that Perceived Usefulness is a strong and significant determinant of Behavioral Intention to Use are consistent with related research. Deng et al. (22) found that perceived value had significant effects on both attitudes toward mobile health services and behavioral intention. When considering physicians as users, Gagnon et al. (21) found that, in all of their proposed relevant models, Perceived Usefulness had a significant influence on Behavioral Intention to Use. Similarly, Hung et al. (29) further investigated if Perceived Usefulness could even indirectly influence Behavioral Intention to Use by positively influencing attitudes. It can be inferred that promoting a positive attitude toward e-health technology can enhance users' adaptation to apps that monitor overuse.

The most interesting new finding concerned the relationship between Perceived Ease of Use and Behavioral Intention to Use. The important point here was that, although this research is congruent with other research reporting that Perceived Ease of Use is a significant determinant of Behavioral Intention to Use (21, 30), the influence was negative rather than positive. This may be considered a contradictory result to some prior research, including Melas et al. who stated that users tend to look for easy tools (19) rather than complex ones. It can be inferred from these results that users will no longer simply show intention to use an app because it is easy to use. That is, having a utility or structure that is too simplistic may hinder the intention to use it, possibly due to lowered credibility that the app is sufficiently professional to take care of one's health. This may be because recent users have increased smartphone adaptability and usage, and that healthcare users have especially become much smarter with using apps than in the past. Consequently, healthcare-related smartphone app services should appeal to the users in terms of their usefulness in order to be accepted as a reliable tool that is expected to contribute to healthcare.

The results indicating that Perceived Security had no significant influence on Behavioral Intention to Use contradict some previous research. Cimperman et al. (23) emphasized that Perceived Security is one of the three key factors that influence acceptance, it can be inferred from our findings that this is not applicable to the logic of accepting smartphone overuse monitoring apps. Ebert et al. (24) also stated that Perceived Security significantly affects acceptance of internet-based mental health interventions. These contradictions may have been caused by the following issues. First, our results could indicate a new approach that, in the case of smartphone overuse monitoring apps, the data security issues do not significantly override their desire to overcome their overuse. Second, Ketelaar (31) mentioned that, in a fundamental sense, the users' earlier position on the adoption curve and their smartphone literacy (which is congruent with discussions held regarding the effect of Perceived Ease of Use on Behavioral Intention to Use) decreases the strength of the connection between privacy concerns and attitude.

Another interesting point is that Resistance to Change had a positive significant effect on Behavioral Intention to Use, which is not consistent with prior research. In terms of smartphone apps vs. electronic medical records, William (32) found that resistance to change had an indirect negative significant effect on Behavioral Intention to Use. However, the author's results may have been different if the direct influence was investigated. Deng et al. (22) also proved that Resistance to Change had a negative and significant effect on the Behavioral Intention to Use mobile services among middle-aged people, which contrasts with our results. Somewhat similar results to Deng et al. were found among older groups of people, in which Resistance to Change had no significant effect on Behavioral Intention to Use. Despite the strong rejection of change, users of healthcare apps would still choose to use them in order to increase their well-being. In addition, considering that our participants were users that were already considerably accustomed to using smartphones (note that, as explained in the methods section, only users who have smartphone usage experience were selected), their characteristics indicate that they are already negative toward Resistance to Change, and that it was only not shown in the statistical results. This is why their Behavioral Intention to Use was positive despite the results. This can also be inferred from the β value, which was the lowest (2.074) of all the statistically significant β values. Lastly and most importantly, Resistance to Change is very possibly influenced the most by the neurophysiological impacts of the emissions from the devices themselves. Therefore, without adequate control, it is not clear what that means relative to the other variables.

Social Norm had a significant effect on Behavioral Intention to Use, which is consistent with prior findings in related fields (21, 23, 24). Additionally, Hsiao and Chen (33) pointed out that social influence was one of the critical factors influencing intention to use. Although a narrower concept, Hung et al. (29) also indicated that co-workers' viewpoints positively influenced intention to use. Therefore, the finding that facilitating the support of the families or friends of the users helps to boost the users' intention to use should be consistently considered from the early education/facilitation stage of the smartphone overuse monitoring system until the end of the intervention.

## Conclusions

The objective of this study was to investigate the factors influencing the users' intention to use a smartphone overuse monitoring app for smartphone overuse prevention and treatment interventions. The findings regarding Perceived Ease of Use influencing Perceived Usefulness, Perceived Usefulness influencing Behavioral Intention to Use, and Social Norm influencing Behavioral Intention to Use were in accordance with previous related research.

The core findings regarding the negative influence of Perceived Ease of Use on Behavioral Intention to Use, Perceived Security's non-significant influence on Behavioral Intention to Use, and Resistance to Change's positive influence on Behavioral Intention to Use were in contrast to some meaningful related research. These can be considered to be unique findings related to the smartphone overuse monitoring app intervention, which opens some discussion that requires future research.

One limitation of this research is that the population did not include adolescents, who are known to be heavy smartphone users who are particularly susceptible to overusing these devices. Another shortcoming is that the neurophysiological impacts of the devices themselves were not adequately controlled.

Future research is being planned that actually applies the findings of this study and implements the SOMS to investigate health-related outcomes. Future research should also include further investigation of the difference in perception between genders regarding the acceptance of the app, since the possibility of a difference has been suggested in previous research (34).

The research results not only identify the critical factors to consider when implementing the SOMS, they are also expected to provide successful mainstream guidelines for future systems or solutions that tackle the issue of smartphone overuse with apps. For example, there are now many apps that are being released that have screen-time monitors. This is a practically useful aspect, especially when considering Apple's CEO Tim Cook's recent quote that even he uses his smartphone too much. Most importantly, our unique points are envisioned to provide intensive insights for broadening knowledge about technology acceptance in the field of e-addictology (35).

## Author Contributions

S-JL designed the study and manuscript. MC collected data and conducted statistical analysis. MR participated in study. D-JK and IC co-corresponded and drove the research project.

### Conflict of Interest Statement

The authors declare that the research was conducted in the absence of any commercial or financial relationships that could be construed as a potential conflict of interest.

## Abbreviations

AGFI: Adjusted Goodness-of-Fit Index
App: Application
AVE: Average Variance Extracted
BIU: Behavioral Intention to Use
CFI: Comparative fit index
CR: Construct Reliability
EMR: Electronic Medical Records
GFI: Goodness-of-Fit Index
ICT: Information and Communications Technology
NNFI: Non-Normed Fit Index
NRF: National Research Foundation of Korea
PEoU: Perceived Ease of Use
PS: Perceived Security
PU: Perceived Usefulness
RC: Resistance to Change
RMR: Root mean Square Residual
SAMS: Smartphone Addiction Management System
SD: Standard Deviation
SN: Social Norm
SOMS: Smartphone Overdependence Management System
TAM: Technology Acceptance Model
UTAUT: Unified Theory of Acceptance and Use Technology.
