# Corrigendum: Factors Affecting User Acceptance in Overuse of Smartphones in Mobile Health Services: An Empirical Study Testing a Modified Integrated Model in South Korea

**DOI:** 10.3389/fpsyt.2019.00259

**Published:** 2019-04-26

**Authors:** Seo-Joon Lee, Mun Joo Choi, Mi Jung Rho, Dai-Jin Kim, In Young Choi

**Affiliations:** ^1^Research Institute of Health Science, Korea University, Seoul, South Korea; ^2^Department of Medical Informatics, The Catholic University of Seoul, Seoul, South Korea; ^3^Department of Biomedicine and Health Sciences, College of Medicine, The Catholic University of Korea, Seoul, South Korea; ^4^Catholic Institute for Healthcare Management and Graduate School of Healthcare Management and Policy, The Catholic University of Korea, Seoul, South Korea; ^5^Department of Psychiatry, Addiction Research Institute, Seoul St. Mary’s Hospital, College of Medicine, The Catholic University of Korea, Seoul, South Korea; ^6^Department of Psychiatry, Seoul St. Mary’s Hospital, College of Medicine, The Catholic University of Korea, Seoul, South Korea

**Keywords:** smartphone overuse, m-Health, TAM, UTAUT, acceptance

In the original article, we neglected to include the funder “Brain Research Program through the National Research Foundation of Korea (NRF) founded by the Ministry of Science, ICT & Future Planning, NRF-2015M3C7A1064796” to all authors.

A correction has therefore been made to the **Acknowledgements**:

“This research was supported by the Brain Research Program through the National Research Foundation of Korea (NRF) founded by the Ministry of Science, ICT & Future Planning, NRF-2014M3C7A1062893 and NRF-2015M3C7A1064796.”

The authors apologize for this error and state that this does not change the scientific conclusions of the article in any way. The original article has been updated.

